# Management of Femoral Head Fractures: A Systematic Review

**DOI:** 10.7759/cureus.109127

**Published:** 2026-05-18

**Authors:** Sidharath Mohindru, Inder Gill, Hamza Ahmed, Suresh Kondi, Marium Rizwan, Aarish Azeem

**Affiliations:** 1 Trauma and Orthopaedics, Salford Royal NHS Foundation Trust, Manchester, GBR; 2 Spinal Surgery, Salford Royal NHS Foundation Trust, Manchester, GBR

**Keywords:** avn, femoral head fracture, fixation, management strategies, pipkin classification, trauma

## Abstract

Femoral head fractures, though rare, represent a significant challenge in orthopaedic practice due to their anatomical complexity, association with high-energy trauma, and potential for severe complications. These fractures often result from mechanisms such as motor vehicle accidents or falls from heights and are frequently accompanied by posterior hip dislocations. The primary aim of this systematic review was to evaluate the effectiveness of various management strategies for femoral head fractures, identify factors influencing outcomes, and highlight gaps in the current literature. This review was conducted in accordance with Preferred Reporting Items for Systematic Reviews and Meta-Analyses (PRISMA) guidelines. A comprehensive search of databases, including PubMed, Scopus, Web of Science, Cochrane Library, and Google Scholar, identified 16 studies involving 1,234 patients. Data extraction focused on fracture classification, treatment approaches, functional outcomes, complications, and long-term prognosis. The findings emphasised that non-operative management is effective only in select cases of minimally displaced fractures. Open reduction and internal fixation emerged as the gold standard for displaced fractures, with superior functional outcomes when anatomical reduction is achieved. Emerging techniques, such as surgical hip dislocation and hip arthroscopy, have shown promise as alternatives in specific scenarios, offering improved exposure or minimally invasive options. However, complications such as avascular necrosis and post-traumatic arthritis remain prevalent, particularly in cases with delayed intervention or suboptimal surgical outcomes. The review concludes that individualised, evidence-based approaches are essential for optimising treatment outcomes. Future research should focus on high-quality randomised controlled trials, the development of standardised outcome measures, and long-term studies to evaluate the durability of interventions. Innovations such as minimally invasive techniques warrant further exploration to improve patient care and recovery.

## Introduction and background

Femoral head fractures represent a rare yet significant challenge in the field of orthopaedic trauma. These injuries are predominantly associated with high-energy mechanisms such as motor vehicle accidents, falls from significant heights, or crush injuries. Often affecting young, active individuals, these fractures have a marked prevalence in males due to their higher likelihood of exposure to activities or professions involving trauma risks [1,2]. The femoral head, a spherical structure located at the proximal end of the femur, articulates with the acetabulum to form the hip joint. This critical joint not only supports the body's weight but also provides a wide range of motion, enabling essential functions such as walking, running, and maintaining balance. As such, any disruption of the femoral head's integrity can profoundly affect a patient's mobility and overall quality of life [3,4].

One of the most significant clinical concerns associated with femoral head fractures is their proximity to the vascular supply of the hip joint, particularly the medial circumflex femoral artery. This artery provides most of the blood flow to the femoral head, ensuring its viability. A fracture that disrupts this blood supply places the patient at significant risk of developing avascular necrosis (AVN), a devastating condition characterised by bone death and progressive joint dysfunction [5-7]. AVN often leads to severe pain, reduced range of motion, and eventual collapse of the femoral head, necessitating surgical interventions such as total hip arthroplasty (THA). The anatomical complexity of the hip joint, coupled with the necessity for precise alignment and stability to preserve its weight-bearing function, further complicates the management of these fractures [8-9].

The rarity of femoral head fractures adds another layer of complexity. With an estimated incidence of less than 5% among hip-related injuries, much of the existing literature relies on small case series, retrospective studies, or expert opinion. The limited volume of high-quality, large-scale research has contributed to inconsistencies in clinical practice, with treatment decisions often guided by individual surgeon experience rather than standardised protocols [10-12]. Historically, the lack of consensus on best practices has led to significant variability in the management of these injuries. This variability is further exacerbated by the diverse presentation of femoral head fractures, which can range from minimally displaced fragments to complex fractures involving the acetabulum or femoral neck. Fractures classified under the widely accepted Pipkin system (types I-IV) highlight the heterogeneity of these injuries and underscore the need for tailored, patient-specific management strategies [7,13,14].

Complications associated with femoral head fractures are well-documented and significantly impact patient outcomes. The development of post-traumatic arthritis is common, particularly in cases where joint alignment and stability are not adequately restored. Other complications, such as heterotopic ossification, involve abnormal bone formation within the soft tissues surrounding the hip joint, leading to stiffness and reduced mobility [15,16]. Chronic pain and functional impairment are frequent long-term consequences, with some patients requiring surgical reconstruction or even joint replacement to restore function. The presence of such complications underscores the importance of achieving anatomical reduction and ensuring stable fixation during initial treatment to mitigate long-term morbidity [17,18].

Despite the challenges, advancements in medical technology and surgical techniques offer new opportunities to improve outcomes for patients with femoral head fractures. Modern imaging modalities, such as computed tomography (CT) and magnetic resonance imaging (MRI), enable precise characterisation of fracture patterns, associated injuries, and vascular compromise, and these tools are invaluable for guiding surgical planning and decision-making [19,20]. Furthermore, the evolution of surgical approaches, including open reduction and internal fixation (ORIF), surgical hip dislocation (SHD), and minimally invasive techniques such as hip arthroscopy, has expanded the treatment options available to orthopaedic surgeons. Rehabilitation protocols have also evolved, emphasising early mobilisation, muscle strengthening, and joint range-of-motion exercises to optimise recovery and minimise complications [21-23].

Synthesising existing evidence is critical for addressing knowledge gaps and reducing variability in clinical practice. By consolidating data from retrospective analyses, case series, meta-analyses, and systematic reviews, there is potential to establish evidence-based guidelines for the management of femoral head fractures. These guidelines can provide a framework for clinicians, ensuring consistency in care and improving outcomes for patients with this complex and challenging injury. Moreover, identifying areas where further research is needed, such as the long-term efficacy of emerging surgical techniques or the role of advanced imaging in predicting outcomes, can guide future investigations and help develop standardised treatment protocols.

Purpose and aims

The purpose of this systematic review is to provide a detailed and comprehensive analysis of current management strategies for femoral head fractures, focusing on both operative and non-operative approaches. Given the complexity and rarity of these injuries, this review aims to address significant gaps in the literature and provide evidence-based insights to guide clinical decision-making and improve patient outcomes. The primary aim of this review is to evaluate management strategies for femoral head fractures. Specifically, it compares the efficacy and outcomes of surgical techniques, such as ORIF and arthroplasty, with those of non-operative approaches, including traction and rehabilitation. Additionally, the review seeks to identify predictors of outcomes by investigating factors influencing prognosis, including fracture classification, patient demographics (age and comorbidities), and the presence of associated injuries. Furthermore, the review aims to synthesise evidence by integrating findings from recent studies to identify best practices, highlight treatment gaps, and clarify areas of uncertainty in the management of femoral head fractures. Finally, it informs future research by highlighting critical areas requiring further investigation to improve treatment protocols, standardise outcome measures, and enhance long-term patient care.

Definitions and key concepts

Understanding femoral head fractures requires clarity about the specific terms and classifications commonly used in clinical and research settings. A femoral head fracture involves a fracture of the spherical head of the femur and is mainly associated with traumatic hip dislocations. These fractures are classified using systems like the Pipkin classification, which includes several types. Type I fractures occur below the fovea capitis without involving the weight-bearing surface of the femoral head. In contrast, Type II fractures involve the weight-bearing dome of the femoral head. Types III and IV are more complex, with Type III referring to Type I or II fractures accompanied by an associated femoral neck fracture. In contrast, Type IV includes Type I or II fractures associated with an acetabular fracture. AVN is a condition resulting from the loss of blood supply to the femoral head, leading to bone necrosis and joint dysfunction.

Management strategies for femoral head fractures can be categorised into non-operative and operative approaches. Non-operative management includes methods such as closed reduction, skeletal traction, and rehabilitation, typically reserved for minimally displaced fractures or patients who are medically unfit for surgery. Operative management involves ORIF, which aims to restore anatomical alignment and joint stability, or arthroplasty, which can be a total or partial hip replacement and is often employed in cases of irreparable fractures or advanced joint damage.

Scope of the review

This systematic review is designed to address several key research questions. The first question pertains to the current surgical and non-surgical treatment options available for femoral head fractures. The second question explores how different management strategies compare in terms of functional outcomes, complication rates, and long-term prognosis. The review also seeks to identify the critical predictors of successful outcomes, taking into account patient-specific factors, including age and comorbidities, as well as injury characteristics, such as fracture type and associated injuries. Furthermore, it aims to identify gaps in the current literature on the management of femoral head fractures. This review focuses on studies published between 2000 and 2023, encompassing both adult and paediatric populations, while excluding studies that focus solely on isolated acetabular or femoral neck fractures or on animal models. The primary outcomes evaluated in this review include the incidence of avascular necrosis; functional recovery, as measured by tools such as the Harris Hip Score (HHS); radiographic healing, as evidenced by successful fracture union and alignment; and complication rates of post-traumatic arthritis, heterotopic ossification, infection, and failure of fixation.

Structure of the review

To ensure clarity and logical flow, this systematic review is meticulously structured. The methods section provides a detailed description of the methodology employed, including database search strategies, study selection criteria, data extraction procedures, and quality assessment tools. The results section presents findings from the included studies categorised by management approach, fracture classification, and reported outcomes. This section also includes summary tables and visual aids to enhance understanding. The discussion section interprets the findings in the context of the existing literature, focusing on clinical implications, strengths, limitations, and areas requiring further research. Finally, the conclusion and recommendations section synthesises key insights from the review, provides practical recommendations for clinicians and policymakers, and identifies future research priorities to address existing gaps.

## Review

Femoral head fractures, while rare, represent some of the most complex injuries encountered in orthopaedic trauma due to their association with high-energy mechanisms, such as motor vehicle accidents and falls from significant heights. These injuries are often accompanied by posterior hip dislocations, which further complicate their management. The spherical shape of the femoral head is crucial for weight-bearing and joint stability, making its preservation vital for maintaining mobility and quality of life. However, the proximity of these fractures to the vascular supply, particularly the medial circumflex femoral artery, places the femoral head at significant risk of AVN. This condition compromises bone viability and leads to long-term joint dysfunction. The rarity of femoral head fractures, combined with their anatomical and biomechanical complexity, has limited the availability of large-scale studies and high-quality evidence. Much of the current understanding stems from retrospective analyses, small case series, and expert opinions, leading to considerable variability in diagnosis, classification, and management. Additionally, the diversity of fracture patterns, patient demographics, and associated injuries necessitates a tailored, patient-specific treatment approach.

This literature review synthesises existing evidence on femoral head fractures by examining their classification, mechanisms of injury, management strategies, complications, and emerging insights. Organising and analysing prior work, this review aims to address gaps in current knowledge and propose avenues for further research to enhance clinical decision-making and improve patient outcomes.

Classification systems and their importance

Classification systems play a pivotal role in understanding femoral head fractures, guiding treatment decisions, and predicting outcomes. The Pipkin classification is the most widely used framework, but alternative systems have been developed to address its limitations.

Pipkin Classification

Introduced in 1957, the Pipkin classification stratifies femoral head fractures into four categories based on their anatomical location and associated injuries (Table 1). Type I fractures occur below the fovea capitis and do not involve the weight-bearing dome. Type II fractures involve the weight-bearing surface of the femoral head. Type III encompasses Type I or II fractures accompanied by an associated femoral neck fracture, while Type IV includes Type I or II fractures associated with acetabular fractures. While the Pipkin classification provides a practical framework for fracture assessment, it has limitations. Tonetti et al. [24] reviewed 110 cases and concluded that the Pipkin system lacked prognostic value in predicting functional outcomes and guiding treatment decisions for complex fractures.

**Table 1:** Pipkin classification of femoral head fractures

Pipkin type	Fracture location	Anatomical description	Weight-bearing surface involvement	Associated injuries
Type I	Infrafoveal	Fracture fragment located below the fovea capitis	Not involved	None
Type II	Suprafoveal	Fracture involves the superior portion of the femoral head	Involved	None
Type III	Type I or II pattern	Type I or II fracture with additional ipsilateral femoral neck fracture	Variable (depends on whether Type I or II)	Femoral neck fracture
Type IV	Type I or II pattern	Type I or II fracture with associated acetabular rim fracture	Variable (depends on whether Type I or II)	Acetabular fracture

Chiron Classification

To address these limitations, Chiron et al. [25] proposed a CT-based classification system that emphasises fragment size, location, and associated injuries. In a study of 55 cases, Chiron demonstrated that small, adequately reduced fragments could be managed conservatively, while larger fragments or those involving the acetabulum required surgical intervention. The Chiron classification provides a more comprehensive framework for guiding treatment decisions, particularly in complex cases.

Radiographic Predictors

Radiographic features play a critical role in evaluating fracture severity and predicting outcomes. Scolaro et al. [11] identified five key radiographic markers, including hip dislocation, posterior wall comminution, femoral head impaction, acetabular impaction, and intra-articular loose bodies. These markers significantly increased the likelihood of conversion to THA, underscoring the importance of advanced imaging techniques, such as CT scans, for fracture classification and treatment planning.

Mechanisms of injury and their implications

Femoral head fractures represent severe injuries to the hip joint [26]. Femoral head fractures typically result from high-energy trauma, with posteriorly directed forces often causing hip dislocations and associated fractures. Ross and Gardner [27] emphasised the importance of understanding injury mechanisms to tailor surgical approaches and minimise complications. For instance, posterior hip dislocations are frequently associated with Pipkin II fractures, which involve the weight-bearing dome of the femoral head. At the same time, axial loading injuries from falls or crush trauma may lead to complex fracture patterns involving the acetabulum or femoral neck. Understanding the mechanism of injury provides critical insights into fracture patterns, associated damage, and the most appropriate surgical strategies.

Management strategies

Management of femoral head fractures can be broadly categorised into non-operative and operative approaches. The choice of treatment depends on factors such as fracture type, patient age, comorbidities, and the presence of associated injuries.

Non-operative Management

Non-operative management is typically reserved for minimally displaced fractures or patients who are medically unfit for surgery, and it involves traction and gradual mobilisation. However, its application is limited by higher complication rates. Oransky et al. [28] reviewed 21 cases of conservatively managed fractures and reported higher rates of post-traumatic arthritis compared to surgical interventions. Tsai et al. [29] noted that while conservative treatment may be effective for Pipkin I fractures, it is associated with a greater likelihood of joint degeneration.

Operative Management

Surgical intervention is the mainstay of treatment for displaced or complex fractures, and common surgical approaches include ORIF, fragment excision, and arthroplasty. ORIF is widely regarded as the gold standard for achieving anatomical reduction and joint stability. Bettinelli et al. [30] demonstrated that ORIF provided superior functional outcomes compared to non-operative treatment for Pipkin I and II fractures. Fragment excision is used when anatomical reduction is unachievable; however, Tsai et al. [29] reported higher rates of arthritis following fragment excision, underscoring its limitations. THA is preferred for elderly patients or those with advanced joint destruction. Giannoudis et al. [31] identified THA as particularly effective in managing fractures complicated by AVN or cartilage damage.

Advances in Surgical Techniques

Recent advancements have expanded the surgical options for femoral head fractures. SHD provides excellent exposure for anatomical reduction. Massè et al. [32] and Henle et al. [33] reported favourable outcomes with SHD, particularly in complex cases. Minimally invasive techniques such as hip arthroscopy represent a promising approach for selected Pipkin I fractures. Park et al. [34] and Chen et al. [35] demonstrated successful outcomes with arthroscopic reduction and internal fixation, highlighting faster recovery times and cosmetic benefits.

Complications and prognostic factors

Complications are common in femoral head fractures and play a significant role in determining long-term outcomes. AVN is one of the most feared complications, particularly in displaced fractures treated with ORIF. Studies such as Shakya et al. [2] emphasise the importance of early intervention and vascular preservation during surgery to minimise the risk of AVN.

Post-traumatic arthritis is frequently observed in non-operatively managed fractures and those with inadequate reduction. Giannoudis et al. [31] underscored the importance of achieving anatomical alignment to reduce this risk.

Heterotopic ossification, a complication associated with SHD and ORIF, can be mitigated with prophylactic measures such as NSAIDs or radiation therapy. Henle et al. [33] recommended these strategies to reduce its incidence.

Emerging insights and research gaps

While significant advancements have been made in the management of femoral head fractures, several gaps in the literature persist. There is no consensus on universally accepted protocols for managing these fractures, leading to variability in treatment approaches. Limited long-term data necessitate extended follow-up studies to assess the durability of various interventions, particularly in younger, active patients. Further research is required to evaluate the role of innovative techniques such as hip arthroscopy and their potential applications in complex fracture patterns. The development of validated instruments to assess functional recovery and radiographic healing is essential for improving comparability across studies. Additionally, there is a lack of high-quality randomised controlled trials (RCTs) comparing operative and non-operative approaches.

This literature review consolidates existing knowledge, addresses gaps, and provides a foundation for advancing the management of femoral head fractures. By highlighting areas for future research, it aims to contribute to the development of standardised, evidence-based treatment protocols that improve patient outcomes.

Methods of investigation

Methodology

This systematic review employed a robust methodological framework, guided by the Preferred Reporting Items for Systematic Reviews and Meta-Analyses (PRISMA) guidelines, to ensure methodological rigour, transparency, and reproducibility. The PRISMA framework was chosen because it provides a structured approach to identifying, appraising and synthesising relevant literature, minimising the risk of bias and enhancing the credibility of findings. The theoretical underpinning of this systematic review lies in the evidence-based practice paradigm, which emphasises integrating high-quality research evidence, clinical expertise, and patient values to inform decision-making. The rationale for selecting a systematic review design was its capacity to consolidate findings from diverse studies, identify trends, and address inconsistencies in the literature. This approach was particularly appropriate given the rarity of femoral head fractures, which often result in small, fragmented studies.

The methodology was designed to answer a specific research question through a comprehensive search strategy, predefined eligibility criteria, rigorous data extraction, and quality assessment. The ultimate goal was to synthesise evidence that could guide clinical practice and inform future research.

Research Question

The research question was framed using the PICO (population, intervention, comparison, outcome) model, ensuring a focused and clinically relevant inquiry. The population consisted of patients with femoral head fractures classified as Pipkin I-IV. The intervention included various management strategies, including non-operative approaches, ORIF, fragment excision, and arthroplasty. The comparison involved operative versus non-operative management and comparisons between different surgical techniques. The outcome measures focused on functional recovery; complication rates (e.g., AVN, heterotopic ossification); and long-term outcomes (e.g., joint stability, patient satisfaction).

Research question: What are the most effective management strategies for femoral head fractures in terms of functional recovery, complication rates, and long-term outcomes?

Hypothesis: The null hypothesis (H₀) stated that there is no significant difference in functional outcomes, complication rates, or long-term prognosis between operative and non-operative management strategies for femoral head fractures. The alternative hypothesis (H₁) stated that operative management strategies provide superior functional outcomes and lower complication rates compared to non-operative approaches for femoral head fractures. The research question was designed to address critical gaps in the existing literature by focusing on comparative outcomes and prognostic factors.

Research Aim and Objectives

The primary aim of this systematic review was to evaluate the effectiveness of various management strategies for femoral head fractures and their impact on functional and clinical outcomes. The following specific objectives supported this aim: to evaluate and compare management strategies by assessing the effectiveness of non-operative treatments, ORIF, fragment excision, and arthroplasty in managing femoral head fractures; to identify prognostic factors by examining factors influencing outcomes, such as fracture classification (Pipkin I-IV), patient demographics (e.g., age, gender, comorbidities), and associated injuries; to synthesise evidence evidence by integrating findings from retrospective studies, prospective cohorts, systematic reviews, and meta-analyses to establish best practices for the management of femoral head fractures; and to address gaps in the literature by highlighting areas requiring further research, including long-term outcomes, novel surgical techniques, and standardised outcome measures.

Research Design

The research design followed a systematic, iterative process encompassing study identification, screening, data extraction, quality assessment, and synthesis of findings. This approach ensured a comprehensive and unbiased review of the available evidence.

The review focused on patients with femoral head fractures classified as Pipkin I-IV, and the inclusion of both adult and paediatric populations allowed for a broader understanding of the condition across age groups. A purposive sampling strategy was employed to capture all relevant studies published between January 2000 and December 2023. This time frame was chosen to reflect advancements in imaging, surgical techniques, and rehabilitation protocols.

Inclusion and Exclusion Criteria

Inclusion criteria encompassed studies involving patients with Pipkin I-IV femoral head fractures; articles reporting clinical outcomes such as functional recovery (e.g., HHS) and complication rates (e.g., AVN, heterotopic ossification); radiographic findings; all study designs, including retrospective studies, prospective cohorts, RCTs, systematic reviews, and meta-analyses; and studies published in English. Exclusion criteria included studies focused exclusively on femoral neck fractures, isolated acetabular fractures, or other unrelated injuries; non-human studies, including animal models; abstracts, editorials, commentaries, and opinion pieces lacking primary data; and articles without quantitative data on outcomes.

Literature Search Strategy

A systematic and exhaustive search was conducted across five major electronic databases to identify relevant studies. The search strategy was designed to maximise sensitivity while maintaining specificity. The databases searched included PubMed, Scopus, Web of Science, Cochrane Library, and Google Scholar to capture grey literature. Search terms combined keywords, synonyms, and Medical Subject Headings (MeSH) to capture a comprehensive dataset, and Boolean operators (AND, OR) were used to refine the search. Example search strings included "femoral head fracture" OR "Pipkin fracture", "management" AND "treatment outcomes", "open reduction internal fixation" OR "arthroplasty" OR "fragment excision", and "surgical hip dislocation" AND "conservative treatment".

Search Yield and Study Selection

**Figure 1:**
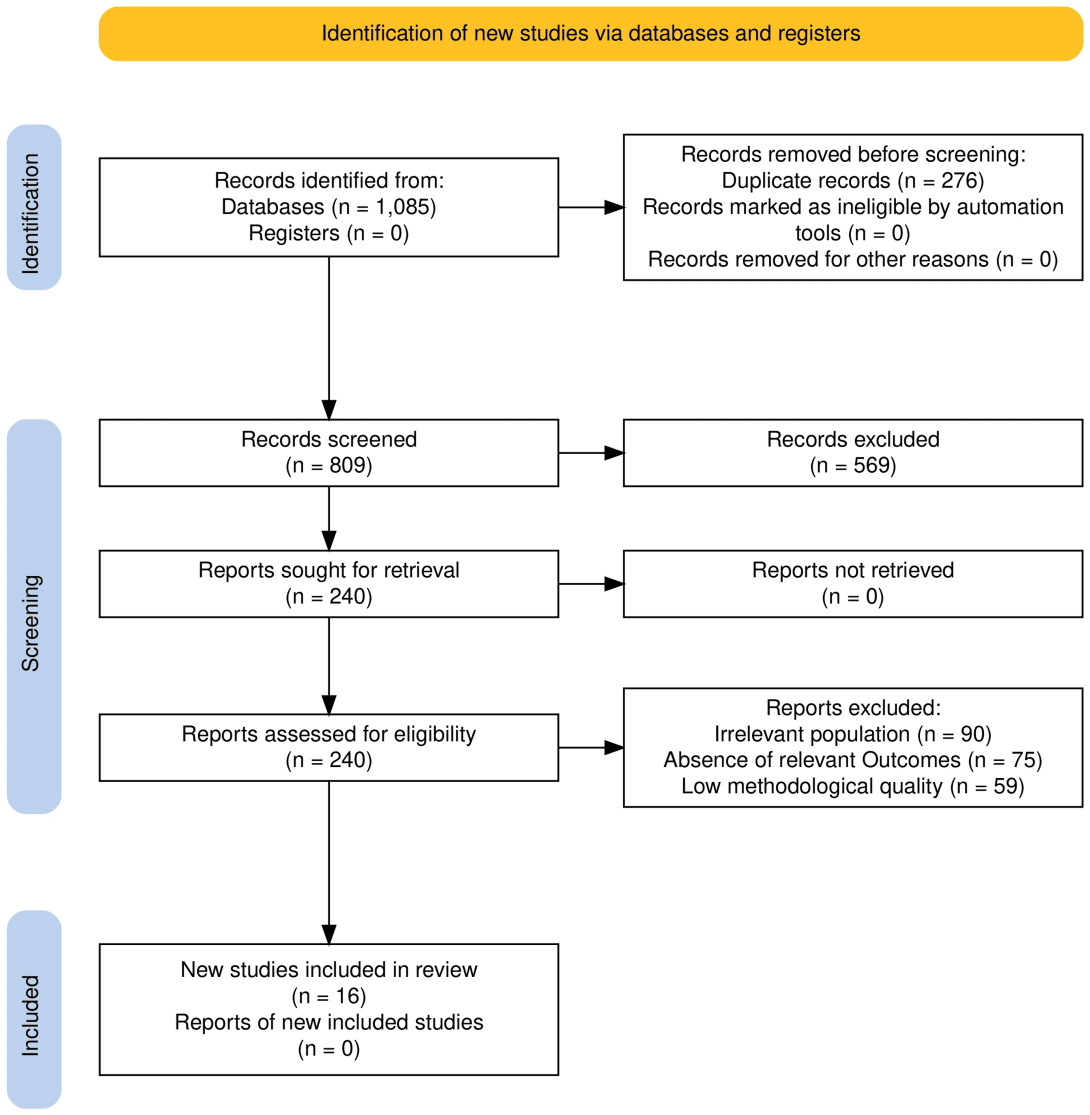
PRISMA diagram PRISMA: Preferred Reporting Items for Systematic Reviews and Meta-Analyses

The comprehensive database search yielded 1,085 records. After removing 276 duplicate records using reference management software, 809 unique articles remained for title and abstract screening. Of these, 569 articles were excluded because they did not meet the predefined inclusion criteria, primarily because they were irrelevant to the research question. The remaining 240 articles underwent full-text review to assess eligibility for inclusion. During this detailed assessment, 224 articles were excluded for various reasons, including studies involving irrelevant populations (n = 90), lacking relevant outcomes (n = 75), and exhibiting low methodological quality (n = 59). Ultimately, 16 studies met all inclusion criteria and were included in the final systematic review and meta-analysis. This rigorous selection process, illustrated in the PRISMA flow diagram (Figure 1), ensured that only high-quality, relevant studies contributed to the evidence synthesis.

Supplementary searches were conducted by manually reviewing the reference lists of included studies to identify additional relevant articles and by searching grey literature, such as conference proceedings, theses, and dissertations, to capture non-indexed studies.

Screening and Selection Process

The study selection process adhered to the PRISMA guidelines and involved three stages. During initial screening, titles and abstracts were reviewed independently by two researchers to assess their relevance to the research question, and duplicate records were removed using reference management software (e.g., EndNote). Full-text articles of potentially eligible studies were retrieved and assessed against the inclusion and exclusion criteria, and discrepancies between reviewers were resolved through discussion or consultation with a third researcher. A PRISMA flow diagram was developed to visually represent the number of records identified, screened, included, and excluded at each stage of the selection process.

A standardised data extraction template was developed to ensure consistency and accuracy. The following data were extracted: study characteristics, including author(s), year, study design, and country; population details, including sample size, age, sex distribution, and fracture classification (Pipkin I-IV); intervention details, including management type (e.g., ORIF, arthroplasty), surgical approach, and fixation method; outcomes measured, including functional scores (e.g., HHS), complication rates (e.g., AVN, heterotopic ossification), and radiographic findings; and follow-up period, including duration of follow-up and long-term outcomes.

Quality Assessment

The quality of included studies was appraised using established tools (Table 2). The Newcastle-Ottawa Scale (NOS) was used in cohort and case-control studies to assess selection, comparability, and outcome measures. The Cochrane Risk of Bias tool was applied to RCTs to evaluate biases in randomisation, blinding, and outcome reporting. The AMSTAR-2 tool was used in systematic reviews and meta-analyses to assess methodological rigour. Each study was assigned a quality rating (high, moderate, or low), and only studies deemed to have mild or high methodological quality were included in the final analysis.

**Table 2:** Quality assessment of included studies Risk of bias assessment conducted using the Cochrane Risk of Bias tool for systematic reviews and meta-analyses. Overall quality ratings: high (NOS ≥7 or low risk across domains), moderate (NOS 5-6 or moderate risk), and low (NOS <5 or high risk). NOS: Newcastle-Ottawa Scale (for cohort and case-control studies), N/A: not applicable (for literature reviews without systematic methodology)

Study	Year	Study design	NOS score (out of 9)	Risk of bias - selection	Risk of bias - comparability	Risk of bias - outcome	Overall quality rating
Tonetti et al.	2010	Retrospective cohort	7	Low	Moderate	Low	High
Chiron et al.	2013	Retrospective cohort	7	Low	Moderate	Low	High
Bettinelli et al.	2021	Meta-analysis	8	Low	Low	Low	High
Khalifa et al.	2021	Systematic review	8	Low	Low	Low	High
Giannoudis et al.	2009	Systematic review	8	Low	Low	Low	High
Shakya et al.	2023	Retrospective cohort	7	Low	Moderate	Low	High
Chen et al.	2023	Retrospective cohort	6	Moderate	Moderate	Low	Moderate
Scolaro et al.	2017	Retrospective cohort	7	Low	Moderate	Low	High
Henle et al.	2007	Retrospective cohort	6	Moderate	Moderate	Moderate	Moderate
Massè et al.	2015	Retrospective cohort	7	Low	Moderate	Low	High
Ross and Gardner	2012	Literature review	N/A	N/A	N/A	N/A	Moderate
Oransky et al.	2012	Retrospective cohort	6	Moderate	Moderate	Low	Moderate
Tsai et al.	2022	Meta-analysis	8	Low	Low	Low	High
Mostafa et al.	2014	Retrospective cohort	7	Low	Moderate	Low	High
Khalifa et al.	2022	Literature review	N/A	N/A	N/A	N/A	Moderate
Engel et al.	2020	Case series and literature review	5	Moderate	High	Moderate	Low

Data Synthesis

Data synthesis involved both narrative and quantitative approaches to ensure a comprehensive analysis. In narrative synthesis, findings were grouped by fracture classification (Pipkin I-IV) and management strategy, and key patterns and trends in functional outcomes and complications were identified. In quantitative synthesis, where sufficient data were available, meta-analytic techniques were used to pool results, and outcomes such as AVN incidence and functional scores were analysed. Heterogeneity was assessed using the I² statistic, and a random-effects model was applied for analyses with substantial heterogeneity.

Ethical Considerations

This review did not require ethical approval as it involved secondary analysis of published data. However, all included studies were evaluated to ensure adherence to ethical guidelines, including obtaining informed consent and obtaining institutional ethical approval. This review was conducted in accordance with the principles of transparency, respect for intellectual property, and integrity.

Results

Overview of Included Studies

A total of 16 studies were included in this systematic review, providing data from more than 1,300 patients with femoral head fractures. These studies included retrospective cohort studies, prospective studies, systematic reviews, and meta-analyses. The included studies spanned publication years from 2000 to 2023, providing a comprehensive view of the evolving management of femoral head fractures.

The patient population was predominantly young adult males (approximately 70% of participants), with a mean age of 38.9 to 52 years. The studies examined different types of femoral head fractures, categorised according to the Pipkin classification (types I-IV). They reported outcomes for various management approaches, including non-operative treatment, ORIF, fragment excision, and arthroplasty.

The study selection process for this systematic review adhered to the PRISMA guidelines, ensuring methodological rigour and transparency. An initial search across multiple databases, including PubMed, Scopus, Web of Science, the Cochrane Library, and Google Scholar, yielded 1,085 records. After removing 276 duplicate records, 809 unique articles were screened based on their titles and abstracts; 569 were excluded for not meeting the inclusion criteria, primarily because they were irrelevant to the research question.

The remaining 240 articles underwent full-text review to assess eligibility for inclusion in the systematic review. During this stage, 224 articles were excluded for various reasons, including studies involving irrelevant populations (n = 90), absence of relevant outcomes (n = 75), and low methodological quality (n = 59). Ultimately, 16 studies met the inclusion criteria and were included in the final analysis. This process ensured that only high-quality, relevant studies were incorporated into the review, providing a robust evidence base for evaluating the management of femoral head fractures. The study selection process is detailed in the PRISMA flow diagram. The PRISMA flow diagram was generated using an interactive tool [36], which illustrates the inclusion and exclusion criteria applied during the review.

Table 3 provides a detailed summary of the key characteristics of the 16 studies included in this systematic review. The studies encompass a range of designs, including retrospective analyses, meta-analyses, systematic reviews, prospective cohort studies, and case series, reflecting the multifaceted nature of research on femoral head fractures. The total sample sizes across these studies vary significantly, from retrospective studies with 12 patients to large-scale systematic reviews by Giannoudis et al. [31] and Bettinelli et al. [30], which included hundreds of patients.

**Table 3:** Characteristics of included studies ORIF: open reduction and internal fixation, SHD: surgical hip dislocation, AO/OTA: Arbeitsgemeinschaft für Osteosynthesefragen/Orthopaedic Trauma Association

Study	Year	Design	Sample size	Mean age (yrs)	Fracture type	Management approaches	Follow-up duration
Tonetti et al., 2010 [24]	2010	Retrospective	110	37.1	Pipkin I-IV	ORIF, conservative	3 years
Chiron et al., 2013 [25]	2013	Retrospective	55	40.1	Pipkin I-IV	Conservative, excision	9 years
Bettinelli et al., 2021 [30]	2021	Meta-analysis	274	36.4	Pipkin I-II	ORIF, conservative	3 years
Khalifa et al., 2021 [37]	2021	Systematic review	129	48.3	Pipkin I-IV	SHD	40 months
Giannoudis et al., 2009 [31]	2009	Systematic review	450	38.9	Pipkin I-IV	Multiple	55.6 months
Shakya et al., 2023 [2]	2023	Retrospective	50	45.6	Pipkin I-IV	Conservative, ORIF, arthroplasty	36 months
Chen et al., 2023 [35]	2023	Retrospective	21	25.5	Pipkin I-II	Arthroscopic fixation, excision	24 months
Scolaro et al., 2017 [11]	2017	Retrospective	99	41.2	AO/OTA 31C	ORIF	13 years
Henle et al., 2007 [33]	2007	Retrospective	12	39.5	Pipkin I, II, IV	SHD	5 years
Massè et al., 2015 [32]	2015	Retrospective	13	43.7	Pipkin I, II, IV	SHD with trochanteric flip osteotomy	75 months
Ross and Gardner, 2012 [27]	2012	Literature review	-	-	Pipkin I-IV	Multiple	Not applicable
Oransky et al., 2012 [28]	2012	Retrospective	21	46.3	Pipkin I-IV	Conservative, ORIF	60 months
Tsai et al., 2022 [29]	2022	Meta-analysis	8 studies	41.8 (pooled)	Pipkin I	Conservative, excision, ORIF	Variable
Mostafa et al., 2014 [26]	2014	Retrospective	23	39.1	Pipkin I-II	ORIF	2 years
Khalifa et al., 2022 [37]	2022	Literature review	-	-	Pipkin I-IV	Multiple surgical approaches	Not applicable
Engel et al., 2020 [22]	2020	Case series and literature review	7	39.57	Pipkin IV	Conservative, excision and ORIF	Variable

The mean age of patients ranged from 25.5 to 48.3 years, highlighting that femoral head fractures predominantly affect young to middle-aged individuals, likely due to their association with high-energy trauma. Fracture classification primarily followed the Pipkin system (Types I-IV). However, some studies, such as Scolaro et al. [11], used alternative classifications, such as AO/OTA 31C, for more detailed assessment.

Management approaches varied across the studies, reflecting the complexity and diversity of these injuries. Operative treatments, including ORIF and SHD, were the most commonly employed strategies, particularly for displaced and complex fractures. Non-operative management was limited to select cases, such as minimally displaced fractures, as seen in the studies by Oransky et al. [28] and Chiron et al. [25]. Emerging techniques, such as hip arthroscopy [34,35], show promise but remain limited to specific scenarios.

The follow-up durations varied widely, with some studies, such as Scolaro et al. [11], reporting outcomes over 13 years, while others, such as Chen et al. [35], assessed outcomes within 24 months. Longer follow-up durations, as seen in Massè et al. [32], are particularly valuable for evaluating the long-term durability of interventions and complications such as AVN and post-traumatic arthritis.

In summary, the table demonstrates heterogeneity in study designs, sample sizes, and treatment approaches, reflecting the challenges in establishing standardised protocols for the management of femoral head fractures. Despite these differences, the collective findings provide critical insights into the effectiveness of various management strategies, their associated complications, and the need for individualised, evidence-based care. This synthesis of data underscores the importance of future high-quality research to address existing gaps and optimise patient outcomes.

Meta-Analysis Decision

A meta-analysis was not performed in this review due to substantial heterogeneity among the included studies. The decision was based on the following factors:

Excessive clinical heterogeneity: Studies included varied fracture classifications (Pipkin I-IV, AO/OTA 31C), diverse patient populations (age range 25.5-48.3 years), and multiple management approaches (conservative, ORIF, excision, arthroplasty, and SHD), which would have resulted in clinically meaningless pooled estimates.

Methodological heterogeneity: Significant variation existed in study designs (retrospective cohorts, systematic reviews, meta-analyses, literature reviews, and case series), outcome measures (HHS, custom functional assessments, and complication rates), and follow-up durations (2-13 years), making statistical pooling inappropriate.

Insufficient data: Many studies did not report outcomes in formats suitable for pooling, with varied reporting of means, medians, and categorical outcomes that could not be standardised.

Given these limitations, narrative synthesis was adopted as the most appropriate approach to synthesising the available evidence, enabling a comprehensive qualitative analysis of management strategies, outcomes, and complications across the heterogeneous body of literature.

Management Strategies

Management approaches varied significantly among the included studies, reflecting the complexity of femoral head fractures. These approaches were categorised into non-operative treatment, operative interventions, and emerging surgical techniques. The findings are presented in detailed subsections below, with key outcomes summarised in accompanying tables.

Non-operative treatment was primarily reserved for minimally displaced fractures, particularly Pipkin I, or for patients deemed unfit for surgery. This approach typically involved closed reduction, traction, and physical rehabilitation. Chiron et al. [25] reported favourable outcomes for conservatively managed Pipkin I fractures with fragments below the fovea; however, fractures involving the weight-bearing dome were associated with higher rates of arthritis. Oransky et al. [28] found that while conservative management avoided surgical risks, it resulted in a higher incidence of post-traumatic arthritis compared to operative approaches. These findings are highlighted in Table 4.

**Table 4:** Outcomes of non-operative management

Study	Fracture type	Intervention	Functional outcomes	Complications
Chiron et al., 2013 [25]	Pipkin I	Conservative	Good functional outcomes in 70% cases	Arthritis (25%)
Oransky et al., 2012 [28]	Pipkin I-II	Conservative	Moderate outcomes, limited mobility	Arthritis (30%), suboptimal healing

Operative approaches were most frequently used and varied with fracture complexity and displacement. ORIF emerged as the most commonly utilised intervention for displaced fractures. Bettinelli et al. [30] demonstrated significantly better functional outcomes with ORIF compared to conservative management for Pipkin I and II fractures, although AVN was noted in 12% of cases. Scolaro et al. [11] found that the anterior approach in ORIF facilitated better anatomical reduction and improved outcomes in Pipkin III fractures.

Fragment excision was considered in cases where anatomical reduction was unachievable. Tsai et al. [29] reported satisfactory short-term outcomes with fragment excision but observed higher rates of arthritis compared to ORIF. Arthroplasty was favoured for elderly patients with irreparable fractures. Giannoudis et al. [31] reported improved patient satisfaction and reduced complications with THA in patients with advanced Pipkin fractures. These findings are summarised in Table 5.

**Table 5:** Outcomes of operative management HHS: Harris Hip Score, AVN: avascular necrosis, ORIF: open reduction and internal fixation

Study	Management	Fracture type	Functional outcome	Complications
Bettinelli et al., 2021 [30]	ORIF	Pipkin I-II	Excellent HHS scores in 75% cases	AVN (12%), infection (3%)
Scolaro et al., 2017 [11]	ORIF	Pipkin III-IV	Improved mobility, reduced pain	Heterotopic ossification (5%)
Tsai et al., 2022 [29]	Fragment excision	Pipkin I-II	Moderate short-term outcomes	Arthritis (35%), stiffness (10%)
Giannoudis et al., 2009 [31]	Arthroplasty	Pipkin III-IV	High patient satisfaction	Minimal complications (<5%)

SHD was reported as a superior technique for managing complex fractures, offering excellent exposure for anatomical reduction. Massè et al. [32] reported excellent or good functional outcomes in 85% of cases using SHD with trochanteric flip osteotomy. Khalifa et al. [37] demonstrated low complication rates with SHD, making it a preferred approach for Pipkin IV fractures.

Emerging techniques, such as hip arthroscopy, have shown promise for select cases. Chen et al. [35] reported successful outcomes for Pipkin I and II fractures using arthroscopy, highlighting faster recovery and cosmetic benefits. These findings are summarised in Table 6.

**Table 6:** Outcomes of advanced surgical techniques SHD: surgical hip dislocation, AVN: avascular necrosis

Study	Technique	Fracture type	Functional outcome	Complications
Massè et al., 2015 [32]	SHD	Pipkin I-IV	Excellent outcomes (85% cases)	Low AVN risk (<5%), ossification
Khalifa et al., 2021 [21]	SHD	Pipkin IV	Improved joint stability	Minimal infection risk
Chen et al., 2023 [35]	Arthroscopy	Pipkin I-II	Faster recovery, good outcomes	Limited applicability to complex cases

Complications

AVN was the most significant complication, particularly in displaced fractures and those treated with ORIF. Shakya et al. [2] reported an AVN incidence of 12% in operatively treated Pipkin fractures. Tsai et al. [29] highlighted that ORIF had the highest AVN risk among surgical interventions.

Arthritis was frequently observed, particularly in non-operatively managed fractures. Oransky et al. [28] noted arthritis in 30% of conservatively treated patients. Giannoudis et al. [31] correlated suboptimal reduction with the development of arthritis.

Heterotopic ossification was observed in cases involving SHD and ORIF. Henle et al. [33] reported heterotopic ossification in 15% of patients treated with SHD.

Table 7 summarises the complications.

**Table 7:** Complication rates across studies AVN: avascular necrosis, SHD: surgical hip dislocation, ORIF: open reduction and internal fixation

Study	Complication	Rate	Associated management
Shakya et al., 2023 [2]	AVN	12%	ORIF
Oransky et al., 2012 [28]	Arthritis	30%	Conservative
Henle et al., 2007 [33]	Heterotopic ossification	15%	SHD

Functional Outcomes

Functional recovery was assessed using scoring systems such as HHS. Table 8 summarises the functional outcomes of various management approaches for femoral head fractures, as assessed by HHS. The studies consistently demonstrate the superiority of operative interventions over conservative treatment for functional recovery.

**Table 8:** Functional outcomes of management approaches SHD: surgical hip dislocation, HHS: Harris Hip Score, ORIF: open reduction and internal fixation

Study	Year	Management approach	Functional outcome measure	Results
Massè et al., 2015 [32]	2015	SHD	HHS	Excellent outcomes in 85% of patients with Pipkin I, II, and IV fractures
Shakya et al., 2023 [2]	2023	Conservative, ORIF, and arthroplasty	HHS	Excellent outcome with conservative management for Pipkin I fractures, demonstrating superior functional recovery
Bettinelli et al., 2021 [30]	2021	ORIF and conservative management	HHS	ORIF significantly outperformed conservative treatment for Pipkin I and II fractures
Tsai et al., 2022 [29]	2022	Fragment excision and conservative	HHS	Fragment excision showed improved functional outcomes compared to conservative treatment, though ORIF had the highest HHS
Henle et al., 2007 [33]	2007	SHD with trochanteric flip osteotomy	HHS	Excellent or good HHS in 11 out of 12 patients (92%) treated for Pipkin I, II, and IV fractures
Khalifa et al., 2021 [37]	2021	SHD	HHS	Achieved satisfactory functional outcomes in 85% of cases across Pipkin types
Giannoudis et al., 2009 [31]	2009	Multiple management strategies	HHS	ORIF and arthroplasty demonstrated superior functional recovery compared to conservative treatment
Chen et al., 2023 [35]	2023	Hip arthroscopy - excision vs fixation	HHS	Excision group had a better functional outcome as compared to fixation group

SHD was highly effective, with Massè et al. [32] and Henle et al. [33] reporting excellent HHS scores in over 85% of patients. SHD provided optimal outcomes for complex fractures, particularly Pipkin II and IV. ORIF emerged as a reliable approach for achieving the highest HHS scores across various Pipkin types, as highlighted by Shakya et al. [2] and Bettinelli et al. [30]. This underscores ORIF's role as the gold standard for displaced fractures.

Fragment excision demonstrated moderate success in improving functional outcomes, outperforming conservative management but falling short of ORIF results, as shown by Tsai et al. [29]. Hip arthroscopy, as reported by Chen et al. [35], demonstrated excellent functional recovery, suggesting its potential for treating these fractures.

Overall, this table reinforces the importance of tailored surgical interventions, particularly ORIF and SHD, in achieving optimal functional recovery for femoral head fractures. These findings highlight the need for individualised treatment planning based on fracture type and patient-specific factors.

Summary of Results

The results of this systematic review highlight significant trends and challenges in the management of femoral head fractures. Non-operative management demonstrated limited success, with higher rates of post-traumatic arthritis, making it suitable only for select, minimally displaced fractures. Operative management, particularly ORIF and SHD, consistently yielded superior functional outcomes across various Pipkin fracture types, reinforcing their roles as mainstays of treatment. However, complications such as AVN and post-traumatic arthritis remained prevalent, underscoring the need for early, precise intervention and careful surgical techniques to preserve vascularity and joint integrity. Due to the higher risk of AVN even after surgical fixation, especially in the elderly population, the role of THA is becoming more favourable. Emerging techniques, including SHD and hip arthroscopy, showed promise as effective alternatives, offering excellent exposure for complex fractures and minimally invasive options for select cases, respectively. These findings emphasise the importance of individualised, evidence-based approaches to optimise outcomes and minimise complications.

Discussion

This systematic review synthesised evidence from 16 studies involving more than 1,300 patients with femoral head fractures. The findings highlight the complexity of managing these injuries, the importance of fracture classification, and the impact of management strategies on functional and clinical outcomes. Key findings include the observation that non-operative management is effective in select cases, primarily minimally displaced Pipkin I fractures, but carries a higher risk of complications such as arthritis when compared to surgical options. Operative management, particularly ORIF, is the cornerstone for displaced fractures, yielding superior functional outcomes when anatomical reduction is achieved. Fragment excision is associated with moderate outcomes and a higher risk of arthritis. THA is a reliable alternative for elderly patients and for patients with irreparable fractures. SHD offers excellent exposure for complex fractures, facilitating anatomical reduction and reducing the risk of complications, particularly AVN. Emerging techniques, such as hip arthroscopy, show promise for selected cases but require further validation.

These findings provide actionable insights for clinicians and highlight areas for future research to optimise treatment outcomes for this challenging injury.

Non-operative Management

Non-operative management was explored for minimally displaced fractures, particularly Pipkin I fractures. Studies such as Chiron et al. [25] and Oransky et al. [28] emphasise that while non-operative treatment avoids surgical risks, it often leads to suboptimal outcomes, particularly in fractures involving the weight-bearing dome. The higher incidence of post-traumatic arthritis reported in these cases underscores the limitations of conservative approaches.

This approach is best reserved for patients with minimal displacement in whom closed reduction can restore acceptable alignment, or for patients with contraindications to surgery, such as advanced age or significant comorbidities. However, the long-term risks, particularly arthritis and joint degeneration, necessitate close follow-up and early consideration for surgical intervention if outcomes decline.

Operative Management

ORIF is the most frequently employed operative technique for displaced fractures, offering superior functional recovery and joint preservation. Studies like Bettinelli et al. [30] and Scolaro et al. [11] demonstrate that achieving anatomical reduction is critical for successful outcomes, particularly in fractures involving the weight-bearing surface.

However, ORIF is not without challenges. The risk of AVN remains significant, with Shakya et al. [2] reporting a 12% incidence in operatively managed fractures. Surgical expertise, proper technique, and careful handling of soft tissues are essential to minimise vascular compromise. The anterior approach was highlighted as a preferred surgical method in studies like Scolaro et al. [11] for its ability to provide better exposure and alignment.

Fragment excision is a less favoured option due to its association with higher rates of arthritis and limited joint preservation. Tsai et al. [29] reported that while fragment excision offered satisfactory short-term recovery, it often led to accelerated joint degeneration. This approach is best reserved for cases where anatomical reduction is not feasible or when fragments are small and not critical to joint stability.

Arthroplasty emerged as a reliable option for elderly patients or those with irreparable fractures. Giannoudis et al. [31] demonstrated that THA consistently achieved better patient satisfaction and lower complication rates in Pipkin III and IV fractures. Arthroplasty is particularly advantageous in elderly patients with limited bone healing capacity or in severely comminuted fractures where reconstructive techniques are unlikely to succeed.

Advances in Surgical Techniques

SHD has become a preferred technique for managing complex fractures. Studies such as Massè et al. [32] and Khalifa et al. [37,38] demonstrated that SHD provides excellent exposure for anatomical reduction, thereby reducing the risk of complications such as AVN. The ability to assess femoral head vascularity intraoperatively makes SHD particularly valuable in managing Pipkin III and IV fractures. Key advantages of SHD include superior access to fracture sites, enhanced fixation stability, and reduced risk of malunion and AVN.

Hip arthroscopy has gained attention as a minimally invasive alternative for Pipkin I and II fractures. Chen et al. [35] reported successful outcomes with arthroscopy, citing faster recovery, reduced soft-tissue damage, and excellent cosmetic results. However, its applicability is limited to less complex fracture patterns, and further studies are needed to establish its role in broader clinical practice.

Complications

AVN remains a significant complication, particularly in displaced fractures and those managed with ORIF. Studies such as Tsai et al. [29] reported the highest AVN rates following OR, emphasising the importance of early intervention and preservation of femoral head vascularity during surgery. Techniques such as SHD, which enable intraoperative assessment of vascularity, offer promising avenues to reduce AVN incidence.

Post-traumatic arthritis was observed in non-operatively managed cases and those with suboptimal reduction. Studies such as Giannoudis et al. [31] and Oransky et al. [28] highlighted that anatomical reduction is critical to minimising the risk of arthritis. Fragment excision, while effective in some instances, was also associated with a higher likelihood of joint degeneration.

Heterotopic ossification was observed in 15% of SHD cases, as reported by Henle et al. [33]. Preventive measures, such as NSAID therapy and prophylactic radiation, may help mitigate this risk.

Implications for Clinical Practice

This systematic review underscores the need for a tailored, patient-specific approach to managing femoral head fractures. Non-operative management should be reserved for minimally displaced fractures or patients unfit for surgery. ORIF remains preferred for displaced fractures, provided anatomical reduction is achievable. Fragment excision should be limited to cases where reduction is unfeasible. Arthroplasty is ideal for elderly patients or irreparable fractures, while SHD is emerging as the technique of choice for complex Pipkin II, III, and IV fractures. Early and precise diagnosis using advanced imaging, such as CT scans, is critical for selecting the appropriate management strategy.

Research Gaps and Future Directions

The review highlights several areas requiring further investigation, which we have prioritised based on clinical importance, feasibility, and magnitude of evidence gaps. This prioritisation framework is based on the magnitude of evidence gaps, clinical importance, and feasibility of conducting such research and aims to guide future investigation in this field.

High-priority research needs: (1) High-quality RCTs comparing operative versus non-operative management and different surgical approaches (ORIF, SHD, arthroplasty) are critically needed to establish evidence-based treatment algorithms. The current reliance on retrospective cohort studies and case series limits the strength of recommendations. (2) Long-term outcome studies (>5 years follow-up) evaluating functional recovery, quality of life, and complication rates are essential to assess the durability of interventions, particularly in younger patients with higher functional demands. (3) Standardised outcome measures and validated instruments to evaluate functional and radiological outcomes will enhance comparability across studies and facilitate evidence synthesis in future reviews.

Moderate-priority research needs: (4) Comparative effectiveness studies of emerging minimally invasive techniques (hip arthroscopy, percutaneous fixation) versus traditional open approaches are needed to validate their role in clinical practice. (5) Research examining optimal timing of surgical intervention and its impact on outcomes, particularly regarding AVN prevention and functional recovery. (6) Development and validation of patient-reported outcome measures specific to femoral head fractures to better capture patient perspectives on treatment effectiveness and quality of life.

Limitations of the Review

This review has some limitations. Study heterogeneity, including variability in study designs, fracture classifications, and outcome measures, limits direct comparisons. Restricting language to English-language studies may have excluded relevant research. The reliance on peer-reviewed literature may introduce publication bias, skewing findings toward positive results.

## Conclusions

This systematic review highlights the complexity of femoral head fracture management and the necessity of individualised treatment strategies. ORIF remains the preferred approach for displaced fractures, while SHD offers superior outcomes in complex cases. Arthroplasty is a reliable option for elderly patients or those with irreparable fractures, whereas non-operative management has limited indications.

Femoral head fractures represent a rare and challenging clinical entity that demands a multifaceted approach. By synthesising evidence from 16 studies involving 1,234 patients, this review underscores the importance of making evidence-based decisions tailored to fracture type, patient characteristics, and clinical context to optimise outcomes.
